# Partial Deployment to Save Space for Vessel Cannulation When Treating Complex Aortic Aneurysms with Narrow Paravisceral Lumen Is Also Feasible Using Inner-Branched Pre-Cannulated Endografts

**DOI:** 10.3390/jcm13113060

**Published:** 2024-05-23

**Authors:** Gioele Simonte, Emanuele Gatta, Vincenzo Vento, Gianbattista Parlani, Rachele Simonte, Luca Montecchiani, Giacomo Isernia

**Affiliations:** 1 Vascular and Endovascular Surgery Unit, S. Maria della Misericordia University Hospital, 06132 Perugia, Italyrachelesimonte@gmail.com (R.S.); 2 Vascular and Endovascular Surgery Unit, Ospedali Riuniti di Ancona, 60126 Ancona, Italy; gattaemanuele@libero.it (E.G.); vincenzovento1987@gmail.com (V.V.); luca.montec@gmail.com (L.M.); iserniagiacomo1@gmail.com (G.I.)

**Keywords:** branched endograft, thoracoabdominal aneurysm, endovascular aortic repair, off-the-shelf endograft

## Abstract

**Introduction:** The aim of this paper is to propose a sequential deployment technique for the E-nside off-the-shelf endograft that could potentially enhance target visceral vessel (TVV) cannulation and overstenting in narrow aortic anatomies. **Methods:** All data regarding patients consecutively treated in two aortic centers with the E-nside graft employing the partial deployment technique were included in the study cohort and analyzed. To execute the procedure with partial endograft deployment, the device should be prepared before insertion by advancing, under fluoroscopy, all four dedicated 400 cm long 0.018″ non-hydrophilic guidewires until their proximal ends reach the cranial graft’s edge. Anticipating this guidewire placement prevents the inability to do so once the endograft is partially released, avoiding potentially increased friction inside the constricted pre-loaded microchannels. The endograft is then advanced and deployed in the standard fashion, stopping just after the inner branch outlets are fully expanded. Tip capture is released, and the proximal end of the device is opened. Visceral vessel bridging is completed from an upper access in the desired sequence, and the graft is fully released after revascularizing one or more arteries. Preventing the distal edge of the graft from fully expanding improves visceral vessel cannulation and bridging component advancement, especially when dealing with restricted lumina. **Results:** A total of 26 patients were treated during the period December 2019–March 2024 with the described approach. Procedure was performed in urgent settings in 14/26 cases. The available lumen was narrower than 24 mm at the origin of at least one target vessel in 11 out of 26 cases performed (42.3%). Technical success was obtained in 24 out of 26 cases (92.3%), with failures being due to TVVs loss. No intraoperative death or surgical conversion was recorded, and no early reintervention was needed in the perioperative period. Clinical success at 30 days was therefore 80.7%. **Conclusions:** The described technique could be considered effective in saving space outside of the graft, allowing for safe navigation and target vessel cannulation in narrow visceral aortas, similar to what has already been reported for outer-branched endografts.

## 1. Introduction

The ongoing endovascular revolution for complex aortic repair aims to provide minimally invasive solutions for a broad spectrum of patients, enhancing endograft adaptability and performance in challenging anatomies. In this scenario, thoracoabdominal off-the-shelf devices have been implemented to offer versatility along with immediate availability.

Since these grafts are frequently, if not mostly, used in urgent/emergent settings for patients with severe contraindications for open repair, technical artifices have been widely adopted to improve immediate and mid-term outcomes in challenging settings.

Partial endograft deployment has been described when using outer-branched endografts in narrow aortic lumina. By keeping the distal portion of the device constrained inside its delivery system, it is possible to increase the available intra-aortic space to properly cannulate and bridge visceral vessels in the critical aortic segment located at the level of target artery ostia [1,2].

The following note describes how this maneuver appears to be feasible and valuable also when using the E-nside (Artivion, Hechingen, Germany) endograft, a pre-cannulated inner-branched endoprosthesis fully available in Europe since January 2021.

## 2. Methods

All patients consecutively treated with the E-nside thoracoabdominal off-the-shelf graft utilizing the partial deployment technique until April 2024 in two Italian aortic centers were included in the study cohort.

All data were included in a database after patient consent was obtained to a voluntary, observational, multi-center data collection. Given the retrospective nature of the study, ethical approval was waived. According to the European General Data Protection Regulation, all cases were deidentified with a coding number and clustered into a dedicated electronic database. No funding was obtained from companies or other institutions for this research. Each patient signed a written consent form for anonymous use of data regarding surgery and follow-up for scientific purposes.

### 2.1. Device Characteristics

The E-nside is a multibranched off-the-shelf endograft, consisting of a nitinol structure surrounded by a polyester fabric. It comes with four antegrade inner branches, positioned in a standard configuration, meant for revascularizing the reno-visceral vessels. The endograft is indicated for the treatment of complex aortic aneurisms, i.e., those involving the origin of the renal and the splanchnic vessels. It is rapidly available, as other off-the-shelf devices, eliminating manufacturing time, and allowing for the rapid assessment of patients presenting with time-dependent pathology, symptomatic or ruptured dilatations.

The graft has a “dog bone” shape, with a proximal and a distal cylindrical section. The proximal section is meant to land at the level of a sealing zone, whether this is represented by healthy proximal aorta or a previously positioned thoracic endograft. The distal landing could either directly achieve sealing in the infrarenal aorta or be extended into the iliac arteries using a standard bifurcated graft.

The E-nside is available in four different combinations of proximal–distal diameters (38 or 33 mm for proximal and 30 or 26 mm for distal). The middle portion, harboring the inner branches, has a standard 24 mm diameter.

A series of radiopaque markers is fixed to the endograft fabric to allow for correct orientation and deployment. There are two “E”-shaped references in the proximal portion aiding rotational orientation and five tubular markers all around the proximal edge of the graft.

Additionally, three dot markers surround the outlet of each inner branch, while a radiopaque ring marks each inner branch inlet. These references are used both for height adjustment while delivering the graft and for target vessel cannulation and bridging.

The endograft is packed into a 24 Fr outer dimeter deliver system (8.2 mm overall diameter); therefore, good-quality access vessels are needed for proper advancement and deployment.

The unique graft feature consists of four microcatheters, running side by side with the distal graft’s portion and crossing each inner branch from distal to proximal and external to internal. These microchannels are used to advance a guidewire that is snared coming from an upper access, allowing for fast inner branch cannulation and target vessel bridging.

The proximal row of nitinol springs is fully covered and asymmetrically configured. This reduces the risk of wires/catheters crisscrossing a bare stent eventually hindering the subsequent introducer sheath advancement from above, while allowing for flexibility and adaptability to tortuous anatomies. The diameter of the inner branches meant to bridge splanchnic vessels is 8 mm, while those for the renal vessels measure 6 mm.

### 2.2. Preoperative Evaluation

Endovascular repair with the E-nside was offered to patients deemed to be high risk for open surgery when anatomically suitable (aortic, iliac, and abdominal visceral vessel features) and planned according to patient-specific characteristics.

Decision whether to ask for a custom-made endograft or proceeding using an off-the-shelf device was taken on the basis of the presence of symptoms, signs of rupture, or impending ruptures in computed tomography angiography (CTA) and aneurysm diameter. In the case of large lumen thoracoabdominal aneurysms with adequate endograft adaptability to the actual anatomy to be treated, the E-nside was chosen over a customized approach considering the advantages provided by pre-cannulation.

In all cases, aneurysm morphology and visceral vessel specifics were assessed by thin-slice computed angio-tomography with proper reconstructions performed using dedicated workstations (Aquarius Terarecon, Foster City, CA, USA and OsiriX DICOM viewer, Pixmeo SARL, Geneva, Switzerland).

All the TVVs were evaluated according to the preoperative CTA using postprocessing evaluations. TVV diameter and length were determined such as the choice of the proper bridging stent–graft. All pre-implantation feasibility assessments were performed by a vascular surgical team with proven experience in complex aortic treatment.

### 2.3. Endpoints

The aim of this study was to assess early results obtained using the partial deployment technique while performing complex aortic repair using the E-nside off-the-shelf endograft. Results were evaluated in terms of intraoperative technical success and 30-day clinical success according to the reporting standards for endovascular aortic repair of aneurysms involving the renal–mesenteric arteries [3].

### 2.4. Statistical Analysis

All statistical data analyses were performed with the SPSS Statistics Software (version 26, IBM Corp, Chicago, IL, USA). Continuous data are expressed as mean ± standard deviation when appropriate and categorical variables as frequencies and percentages.

### 2.5. Partial Deployment Technique

The E-nside is the second off-the-shelf endograft available in Europe after the approval of T-Branch (Cook Medical, Bloomington, IN, USA) in 2012. It is available in four different sizes, featuring a pre-cannulation system with four microcatheters passing through each inner branch from external caudal to internal cranial.

These microcatheters facilitate the advancement of 0.018″ dedicated non-hydrophilic guidewires across each inner branch, which are then snared from an upper access. This establishes a femoral/axillary or femoral/brachial through-and-through, over which an introducer sheath, pushed down from above, can be advanced directly into its target inner branch, allowing the operator to immediately cannulate the proper target visceral vessel and deploy a bridging component. This technology was developed not only to save time during inner branch cannulation but also to enhance system stability, stiffness, and pushability once the through-and-through is established.

The standard implantation technique for the E-nside involves sequential guidewire advancement and snaring in the desired sequence of visceral vessel undertaking after full deployment of the endograft at the intended aortic height [4].

**Figure 1:**
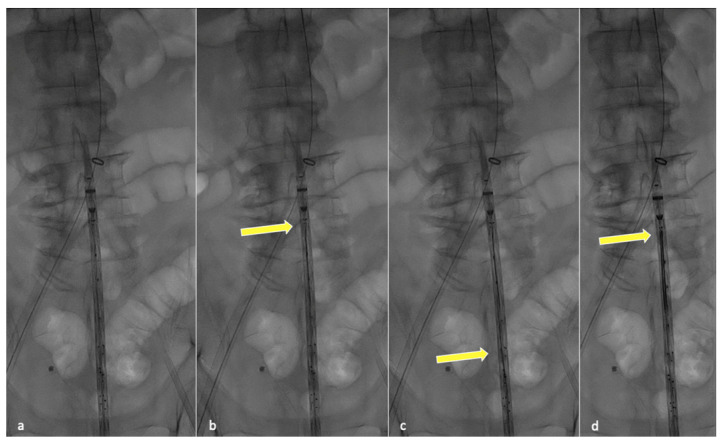
Fluoroscopic check during guidewire anticipated advancement. Graft leaning on patient body (**a**), yellow arrow pointing at the tip of the first positioned guidewire (**b**), second guidewire advancement (**c**) and final results after all guidewires have been correctly advanced (**d**).

In order to perform partial endograft deployment, the device should be prepared before insertion by advancing, under fluoroscopy, all four dedicated 400 cm long 0.018″ non-hydrophilic guidewires through the corresponding microcatheters until their proximal end reaches the cranial graft’s edge (Figure 1).

Anticipating this guidewire placement prevents the inability to do so once the endograft is partially released, avoiding increased friction inside the constricted pre-loaded microchannels.

The endograft is then advanced and deployed in the standard fashion, stopping just after the inner branch outlets are fully expanded. This can be performed either until the celiac trunk and superior mesenteric artery inner branches are released first and then, after bridging, progressively proceeding to the renal vessels, or by directly deploying the graft until just distal to renal branches.

The proximal clasping mechanism is then released, fixing the endograft position, and visceral vessel bridging is completed from the upper access, establishing and taking advantage of the through-and-through. The distal portion of the endograft is completely released after revascularizing one or more target arteries, depending on preoperative planning and the expected hindrance level for target vessel cannulation.

**Figure 2:**
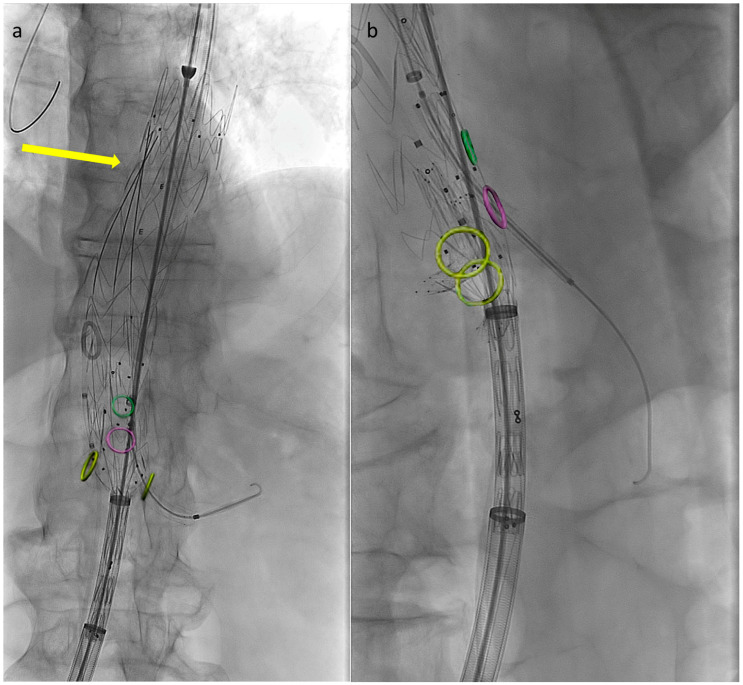
Angiographic images from a case performed using the described technique. Circles indicate visceral vessels origin (positioned using fusion technology). By maintaining the distal endograft constrained into the delivery system the available space for cannulation and bridging components advancement increases. In panel (**a**) the left renal bridging stent has just been released, the arrow is pointing at the three remining pre-positioned 0.018″ pre-cannulated guidewires. In panel (**b**) the superior mesenteric artery bridging stent is correctly positioned and ready to be deployed.

Preventing the distal end of the graft from fully expanding simplifies visceral vessel cannulation maneuvers and bridging component advancement, especially when dealing with restricted lumina (Figure 2). This aspect is even more critical when using an E-nside graft compared to outer-branched devices, given that while the middle portion of the endoprosthesis measures 24 mm in diameter, its distal segment re-expands to 26 or 30 mm with a reverse tapering. The partial deployment approach prevents this distal segment from filling up the inner aortic lumen at the renal artery ostia, a condition dependent on the graft deployment height, potentially leading to target vessel occlusion.

Although not demonstrated yet, the nitinol structure of the E-nside is considered to have much lower radial force compared to the stainless steel T-branch skeleton. This reduces intraluminal conflict after complete graft deployment, providing adequate radial force coming from the bridging components, which in narrow lumen cases, according to the authors’ experience, should be preferentially self-expandable.

## 3. Results

**Figure 3:**
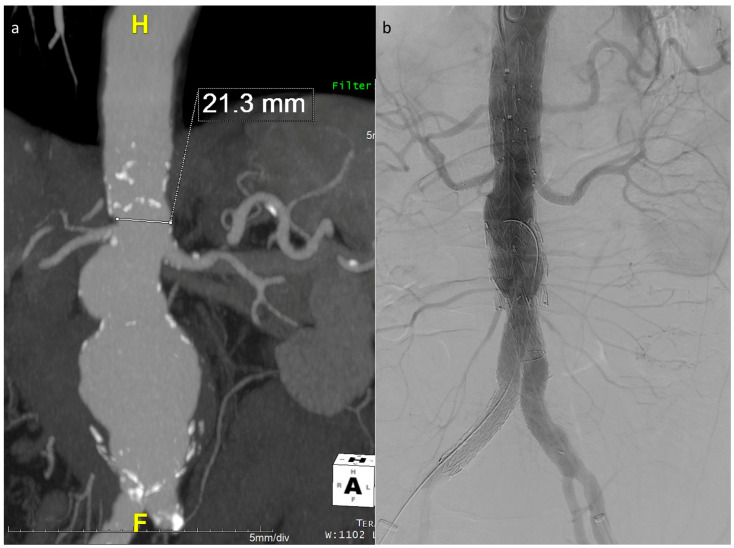
Maximum intensity projection coronal view of the example case in Figure 1, showing a very narrow lumen just above the origin of the renals in a patient with a wide pararenal aneurysm (**a**). Final angiographic control (**b**).

The described technique was used in 26 out of the 40 E-nside cases performed in the two authors’ institutions from January 2020 to March 2024, enabling the treatment of anatomies with an inner aortic lumen down to 20 mm in diameter (Figure 3).

The main indication for using the partial deployment technique was a narrow aortic lumen at the level of one or more than one target visceral vessel. Remaining procedures were performed using the standard deployment sequence, taking advantage of either an upper antegrade access or a retrograde femoral one with steerable sheaths to complete the TVV bridging. 

Most of the patients in the study cohort were male (18/26, 69.2%) with a mean age at the time of the procedure of 77.5 ± 7.9 years.

The procedure was performed in an urgent setting in 14 out of 26 cases, including 2 cases of ruptured aneurysm and 8 cases of symptomatic dilatations.

All patients except one had a degenerative aneurysm. The remaining was a post-dissection type II thoracoabdominal dilatation. Mean aneurysm diameter at the time of the procedure was 66.7 ± 20.3 mm.

Internal aortic diameter measured 36.0 ± 12.5 mm at the origin of the celiac trunk, 36.0 ± 17.0 mm at the origin of the superior mesenteric artery, 31.3 ± 8.7 mm at the origin of the right renal artery, and 30.4 ± 9.0 at the origin of the left renal artery. The available lumen was narrower than 24 mm at the origin of at least one target vessel in 11 out of 26 cases performed (42.3%). Additional details on comorbidities and anatomical features are reported in Table 1.

Aneurysm extent according to the Crawford classification was thoracoabdominal in 20 cases (76.9%) and juxtarenal in 2 cases (7.7%, both treated in an urgent setting because of aneurysm diameter of > 8 cm and abdominal pain). In the remaining cases, the aneurysm involved the origin of at least one renal vessel origin (pararenal according to the Crawford classification).

In most of the cases (73.1%), all TVVs were cannulated and bridged with the graft still distally captured into its delivery system, while in five cases, one vessel was overstented after completing the endograft deployment, and in two cases, this was performed for both splanchnic vessels, while the renal vessels were previously revascularized using the partial deployment approach.

The operating time was 270.0 ± 95.6 min, the fluoroscopy time was 78.5 ± 26.1 min, and 171.3 ± 58.6 mL of iodine contrast media was used.

Technical success was obtained in 24 out of 26 cases (92.3%), with failures being due to TVVs being lost. In the first patient, it was impossible to cannulate the renal vessels (which appeared small and diseased in the preoperative computed tomography), and in another patient, the celiac trunk was not overstented. The corresponding inner branches were embolized in all these cases.

No intraoperative death or surgical conversion was recorded, and no early reintervention was needed in the perioperative period.

Four patients died within 30 days from the index procedure and two developed significant spinal cord ischemia with paraplegia (one of them died on postoperative day 23). Therefore, clinical success at 30 days was 80.7%.

In the control CT scans, performed in all the cases before discharging the patient, no significant bridging stent compression was recorded.

## 4. Discussion

Inner branches represent the latest available configuration for managing target visceral vessel revascularization during complex aortic repair. This approach aims to take advantage of some of the strengths of its historical competitors, allowing for directional ease of cannulation while ensuring a long sealing zone for bridging devices.

Inner branches are preferentially used in patients with narrow aortic lumens as internal cuffs do not suffer for compression risks after completing the procedure, thus arguably improving patency over time [5,6,7].

A narrow aortic lumen represents a common contraindication for branched endovascular repair, creating a scenario where complications such as difficult target visceral vessel cannulation/overstenting or compression of bridging components at the procedure’s conclusion may arise.

The specified minimum inner vascular diameter (aortic lumen) required by the E-nside at the level of visceral vessels origin is 24 mm, while the corresponding value for the T-branch is 25 mm [2,8].

Notably, these measurements are not directly outlined in the Instructions for Use (IFUs) but are derived from manufacturer data or reported studies [2,8,9].

The largest available study as of today reporting outcomes obtained using the E-nside endograft for complex aortic repair analyzed a multicentric cohort of 116 patients treated in 31 different centers. Based on the report by Piazza et al., 15.5% of the patients had an internal aortic diameter measuring less than 25 mm at the TVV origin, a percentage increasing up to 48.3% considering 30 mm as a threshold. The authors report a 98.2% technical success rate and a 90-day mortality rate of 5.2%, with most of the deaths occurring in the subgroup of patients treated in an urgent setting. Interestingly, no differences were found comparing patients with larger aortic lumina with those with an internal diameter of < 25 mm in terms of branch technical success and instability [10].

Bertoglio et al., in 2021, conducted an evaluation on the theoretical anatomic feasibility of endovascular treatment for thoracoabdominal aortic aneurysms (TAAAs) using different off-the-shelf multibranched stent–grafts. Their analysis, based on preoperative CT scans of 268 patients treated for degenerative TAAA repair from 2007 to 2019, revealed that the inner aortic diameter at the level of visceral vessels origin would limit off-the-shelf branched endograft feasibility in 14% of cases for the E-nside and in 17% for the T-branch [8].

This non-negligible limitation is expected to be much higher in cases of juxta/pararenal aneurysms or type Ia endoleaks post previous endovascular aortic repair, conditions in which branched endografts are nowadays increasingly employed both in urgent and elective scenarios [11,12,13,14].

Aortic dissections or post-dissection aneurysms, characterized by a significantly reduced inner diameter of the true lumen, further challenge the standard use of off-the-shelf endografts [15,16,17].

Ferrer et al. recently reported a two-center experience using the T-branch endograft, where 41.7% of patients had an internal aortic diameter measuring less than 25 mm at the paravisceral/pararenal level [14]. Notably, similar outcomes were observed between this narrow-lumen population and the remaining patients. The authors underscore the potential of the T-branch stent graft, when combined with the latest generation of bridging stents, to induce aortic lumen remodeling. This remodeling could reduce the potential encumbrance of the aortic endoprosthesis and bridging stents within strict internal volumes [14].

The challenges posed by narrow aortic lumens highlight the urgent requirement for advanced solutions in endovascular repair. Reported experiences emphasize the significance of considering patient-specific anatomical factors and employing innovative strategies to improve the feasibility and success of endovascular interventions in such challenging scenarios.

The partial endograft deployment technique, initially introduced for outer-branched devices concurrently in two separate papers by Simonte et al. and Malekpour et al. in January 2021, has rapidly gained popularity and is now widely embraced across numerous centers [1,2].

This approach was therefore shifted to inner-branched endografts in the authors’ institutions after satisfactory experiences acquired when using the T-branch. When using outer-branched devices, preventing full endograft distal expansion demonstrated to be useful in avoiding internal conflict in between the main aortic module and bridging components and reducing procedural challenges while allowing for more permissive indications, considering also very narrow aortas treatable with branched grafts.

With these premises, every time a potential difficulty was expected, the partial deployment technique was applied to E-nside repairs.

Based on the authors’ experience, the technique is considered safe for patients requiring a branched endograft repair, particularly in cases with reduced cannulation and navigation space availability outside of the endograft once deployed.

Using inner-branched endografts, this approach could be arguably even more useful than with standard BEVAR. This could be the case since the main graft measures 18 mm at the branch level in the case of the T-branch and 24 mm in the case of the E-nside. This lager diameter could potentially determine the impossibility to cannulate TVV whenever the fully expanded graft fills the entire aortic volume.

While the authors clarify that they do not intend to promote any off-label use, they suggest that the described technique could enhance technical and clinical success if its use becomes necessary.

One potential drawback associated with partial deployment is the increased friction during pre-loaded guidewire advancement, which may occur even if they were properly positioned before endograft insertion. This issue may make it impossibile to further advance the guidewires, resulting in failure to establish and take advantage of the through-and-through, as seen in 4.8% of vessels in authors’ experience. However, in all these instances, direct cannulation from the graft lumen was successfully performed in the standard fashion without encountering difficulties.

To be noticed, the described approach precludes the possibility of completing the procedure taking advantage just of femoral accesses using steerable sheaths. The retrograde approach has been increasingly used in many centers as it provides several advantages (reduced radiation burden, lower stroke risk, improved operating theater ergonomy). Retrograde branch cannulation and bridging is currently employed in the author’s institutions as a first choice in each case partial deployment is not needed [18,19,20].

The advantages provided by partial deployment should therefore be balanced with the impossibility of using retrograde accesses, carefully evaluating the right approach for the specific case.

Utilizing the E-nside in narrow anatomies rather than the T-branch may provide several advantages. A through-and-through access significantly enhances system stability. With the introducer sheath approaching from above, it can easily advance into the inner branches and exit outside of the graft, contributing to gaining space for target vessel cannulation, even when the partially deployed endograft has already occupied the residual luminal volume.

**Figure 4:**
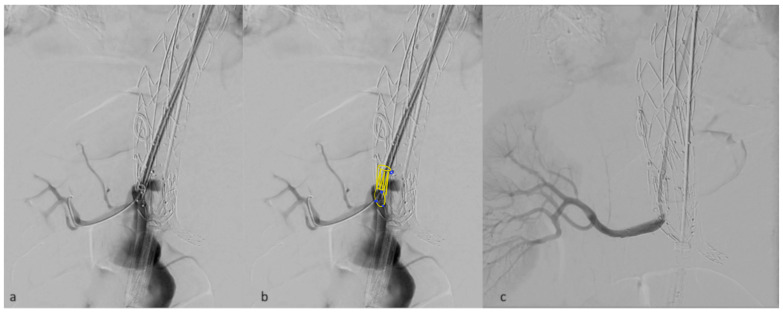
Detail of a right renal inner branch outlet used as a fenestration, deployed just in front of the target vessel ostium (**a**). in panel (**b**) inner branch characteristics have been added in post-processing. Angiographic result after bridging stent deployment (**c**).

Moreover, the vertical outlets of the inner branches in the E-nside allow for their positioning as fenestrations, minimizing the gap to be bridged outside the graft (Figure 4). This configuration prevents the occurrence of parallel paths between the distal graft’s body and the renal bridging stents, a situation that may lead to stent compression, particularly with the T-branch where outer branch outlets are downward-facing.

Additionally, the utilization of inner-branched endografts virtually excludes the risk of branch compression, ensuring the capability to advance catheters and sheaths even in strict anatomies.

Ultimately, the E-nside standard implantation technique is conceived expecting the delivery system to stay in place during the whole visceral bridging phase. For that reason, partial deployment does not prolong leg ischemia.

## 5. Conclusions

The partial deployment technique applied to E-nside endograft aortic repair appears to be effective in saving space outside the graft, enabling safe navigation and target vessel cannulation in narrow visceral aortas. This mirrors the success reported for outer-branched endografts. However, the safety and efficacy of employing the E-nside in such challenging anatomies require further evaluation and establishment.

## Figures and Tables

**Table 1:** Patient characteristics and anatomical features.

Characteristics	*n* (%)
Hypertension	25 (96.2)
Dyslipidemia	16 (61.5)
Diabetes	4 (15.4)
Smoking history	7 (26.9)
Chronic obstructive pulmonary disease	17 (65.4)
Coronary artery disease	16 (61.5)
Previous aortic surgery	13 (50.0)
Thoracoabdominal aneurysm	20 (76.9)
Internal lumen of < 24 mm at least at one TVV origin	11 (42.3)

## Data Availability

Data is contained within the article.
